# Tracking the Flow of Funds in Global Health Security

**DOI:** 10.1007/s10393-019-01402-w

**Published:** 2019-02-28

**Authors:** Rebecca Katz, Ellie Graeden, Justin Kerr, Stephanie Eaneff

**Affiliations:** 1 Georgetown University, 3900 Reservoir Road, NW, 305 SW Med Dent, Washington, D.C., 20057 USA; 2 Talus Analytics, Boulder, CO USA; 3 Talus Analytics, Washington, D.C., USA; 4 Talus Analytics, Boston, MA USA

**Keywords:** Global health security, Financing, Mutual accountability, Mapping dashboard, Tracking dashboard

## Abstract

Countries, philanthropies, and private sector organizations have been actively investing in global health security around the world. However, despite the coordinated approach to funding within the Global Health Security Agenda, there is currently no well-established method to track the commitment and disbursal of funds for global health security from funders to recipients or to identify the activities supported by existing funding initiatives. To address this need, we developed the Global Health Security Tracking Dashboard. This interactive, publicly available, Web-based dashboard maps the flow of funds from funder to recipient and categorizes the target efforts of those funds, allowing users to identify patterns of influence and success in health security funding implementation. The dashboard provides an evidence-based approach for defining targets for future funding by identifying the areas in which funds have not yet been effectively allocated, showcasing successes, and providing a source of information to promote mutual accountability.

**Electronic supplementary material:**

The online version of this article (10.1007/s10393-019-01402-w) contains supplementary material, which is available to authorized users.

## Introduction

With the adoption of revised International Health Regulations (IHR) in 2005, and again in 2018, countries, philanthropies, and other organizations began to explicitly fund efforts designed to increase national-level capacity to prevent, detect, and respond to public health emergencies. Early funding, however, was limited, and as a result, the majority of nations struggled to fully implement the IHR by the 2012 and then 2014 deadlines.^1^ In 2014, the USA joined with the World Health Organization (WHO), Organization for Animal Health (OIE), the United Nations Food and Agriculture Organization (FAO), and other partner nations to launch the Global Health Security Agenda (GHSA). Now with over 60 partner nations, the GHSA was designed to strengthen global capabilities to prevent, detect, and respond to biological threats, and marshal resources to countries that required assistance. The USA alone, through the GHSA, invested $1 billion to support capacity building in 17 nations.^2^

The same year GHSA was launched, the world witnessed the ravages of Ebola in West Africa—a region severely lacking public health and healthcare infrastructure. One of the global lessons learned from the Ebola outbreak was the need for better metrics and processes to assess national capacities and compliance with the IHR.^3^,^4^,^5^ This call for action resulted in the launch of the Joint External Evaluation (JEE) tool in early 2016. The JEE identifies 19 technical areas to measure national core capacities and is the first step in a process designed to identify gaps and build or expand funded programs to strengthen national capacity. This process helps provide a level of standardization that assures funders of the validity of potential investments.

Ensuring adequate funding for global health security is paramount to global efforts to protect against biological threats. However, there are currently limited resources available to track the commitment and disbursals of financial assistance or in-kind support at the project level, across multiple funders, recipients, and core capacities. The resources that do exist include funder-specific efforts,^6^,^7^ disease-specific resources,^8^ and multi-sectoral tracking resources that aggregate funding data across multiple funders, recipients, sectors, and disease areas, but are not specifically focused on health security.^9^,^10^,^11^ The WHO has created a Web site that provides a global overview, but does not allow access to data at the project-specific level or allow users to analyze or assess funding.^12^ Inconsistent tracking and reporting of funding data across funders, recipients, and core capacities makes it difficult to characterize the current status of funding commitments and disbursals, which is essential to identify funding requirements, prioritize future commitments, and develop a compelling case for investments.

Most other major areas of global health, however, have comprehensive funding tracking mechanisms in place, suggesting that tracking global health security should be a tractable goal. Particularly, given recent developments in global health security, the interest in global funders, and the recognized importance of collective action, it is essential to have a mechanism to identify patterns of influence and success in global health security funding implementation. In this paper, we describe the development of the Global Health Security Tracking Dashboard, an interactive, Web-based tool to track and analyze funds for global health security. The tracking dashboard was developed to allow countries, NGOs, and policy experts to better understand who is funding what, and where, in the broad context of global health security, and to support research efforts to identify patterns and best practices in health security.

## Methods

### Data Structure and Ontology

To map the flow of financial transactions in global health security, we defined an ontology to characterize the transfer of committed and disbursed funds or in-kind support from funders to recipients. In this context, funders are defined as committing and/or disbursing financial assistance or in-kind support to a recipient for projects categorized by the Joint External Evaluation (JEE) core capacities they are intended to support. Financial assistance is defined as the transfer of funds directly from funder to recipient, while in-kind support refers to other forms of assistance that do not involve the direct transfer of funds from funder to recipient (e.g., deployment of personnel, support for a GHSA action package). Commitments are official, public obligations from a funder to provide a specified amount of funds or support to the recipient. Disbursements are outgoing funds or support that have been received by the recipient. Both funding and in-kind support are described by the year(s) or planned year(s) of transactions, by the project and core capacities supported, and by whether or not funds were specifically allocated under the GHSA. Additionally, committed and disbursed funds are described by amount and currency of transactions. Funders and recipients include governments, philanthropic organizations, private sector organizations, and other entities.

#### Publically Available Funding Data

The GHS tracking dashboard includes aggregated data from publicly available datasets, and, in the future, will also incorporate data submitted directly through the dashboard. The project-level funding data currently included in the tool reflect data reported in six external sources, including the International Aid Transparency Initiative (IATI), the Article X Compendium, the Ebola Recovery Tracking Initiative, the Nuclear Threat Initiative (NTI) Commitment Tracker, the WHO Contingency Fund for Emergencies, and the 2018 US GHSA Progress and Impact Report with transaction dates or intended transaction dates ranging from January 1, 2014 to June 11, 2018. Additional information on how data were accessed, transformed, and de-duplicated across sources is included in Technical Appendix (available online).

#### Joint External Evaluation Scores

The JEE tool identifies 19 technical areas of health security known as core capacities, each characterized by one to four indicators. After completing the evaluation process, countries receive scores ranging from 1 (*no capacity)* to 5 (*sustained capacity*) for each of 48 indicators identified in the first-edition JEE tool. Evaluation data are made available online by the World Health Organization as part of mission reports and executive summaries and were accessed by the research team and reformatted into the data structure necessary to support subsequent analyses. Additional JEE scores are incorporated into the tool as subsequent evaluations are completed and results are made publicly available.

### Web-based Tracking Dashboard

The GHS tracking dashboard uses custom-built visualizations in JavaScript and d3.js-based interactive graphics to display and communicate the funding data, including a world map that illustrates the global distribution of financial resources and JEE scores, a visual representation of the international GHS funding network, and a set of funder and recipient profiles that contain detailed information on the activities of countries, philanthropies, private sector donors, and other funders. Users may specify their local currency to view results in the format most relevant to them; conversion rates between currencies are calculated based on the United Nations Operational Rates of Exchange as of April 2017.

#### Home Page

The opening page of the GHS tracking dashboard includes a table of top funders and recipients, highlighting those countries, philanthropies, and private sector organizations which have funded or received the greatest amounts of financial resources. A search bar is provided to search for funders or recipients not shown in the overview table, and users may use search or click on the table directly to access funder and recipient profiles. Users may also choose to view information on a map of global financial resources, or as part of an international funding network.

#### Map of Global Financial Resources and JEE

The financial resources and in-kind support each country has provided or received, as well as country-level JEE scores, are visualized on a shaded world map, generated as a d3.js-based choropleth, with filters for time period, in-kind support or direct financial assistance, commitments or disbursements, funder or recipient, core capacity, and whether or not funds/support were allocated under the GHSA. Financial resources are defined as the amount of total funding committed or disbursed and are visualized for all country funders or recipients using a color scale that corresponds to the currency selected by the user. In-kind support is defined based on the number of projects for which support was given or received. In cases where the specific amount of funds or support are not known at the funder–recipient level, shaded, dashed lines are used to indicate that funding or support was given or received, but that the specific amount was not reported.

JEE scores are shown for each country that has completed the Joint External Evaluation process. Scores are visualized using the color conventions used in published JEE reports and are shown as the average score of all indicators corresponding to the core capacities selected in the dashboard’s filters. To identify countries whose needs may still be unmet, a metric of financial resources and needs is calculated as the ratio of total disbursed funds received (on a log scale) to a country’s distance, in points, from a *sustained capacity* score (of 5), as:

$$ 10 + \log_{10} (1 + \;{\text{total}}\;{\text{disbursed}}\;{\text{funds}}\;{\text{received}})/(5.01 - {\text{JEE}}\;{\text{score}}).$$

This metric, which characterizes the ratio of financial resources to need, is used to identify countries on a scale ranging from *needs unmet* to *needs met.* JEE evaluation scores are used as proxy for need, and for a given JEE score, the metric scales according to the logarithm of disbursed funds received. Countries with higher amounts of funding or lower need (as approximated by higher JEE scores) are identified as more likely to have their *needs met*, while countries with lower amounts of funding or higher need are identified as more likely to have their *needs unmet.* The metric is more sensitive to changes in JEE score than to changes in financial resources.

#### International Funding Network

The global flow of funding from funder to recipient is shown as a network using a chord diagram with filters for time period, committed or disbursed funds, and core capacity. Countries and non-country funders are grouped along the outside of the diagram by regions and subregions defined by the United Nations^13^ Statistical Division. Colors correspond to the ratio of amount funded to amount received, with countries or organizations that act primarily as funders shown in blue and those that act primarily as recipients shown in red. Connecting lines show the transfer of funds from funder to recipient and are proportional to the amount of funding commitments or disbursals. Additional details on the funding amount or funders and recipients can be viewed by hovering over each line.

#### Funder and Recipient Profiles

Each funder and recipient represented in the database has an associated funder or recipient profile. Across core capacities, the amount of funds committed or disbursed is shown as a stacked bar chart which displays additional information on funders and recipients if users hover over a segment of the bar chart with their mouse. For each funder or recipient, data may be accessed as a sortable data table of the amount of funds or in-kind support committed and disbursed by project, by funder, by core element, or by core capacity.

#### Data Submission

In addition to the publicly available data accessed by the GHS tracking database, users may also submit data through the online template provided in the dashboard. Users may download and re-upload an Excel-based data reporting template to contribute new or updated commitment or disbursal data. Any data shared by users will be reviewed by the research team for potential duplication before being integrated into the GHS tracking database. In the case that additional information is required to clarify data submitted through the tool, users are asked to provide their name, organization, and contact e-mail address when submitting data.

## Results

**Figure 1:**
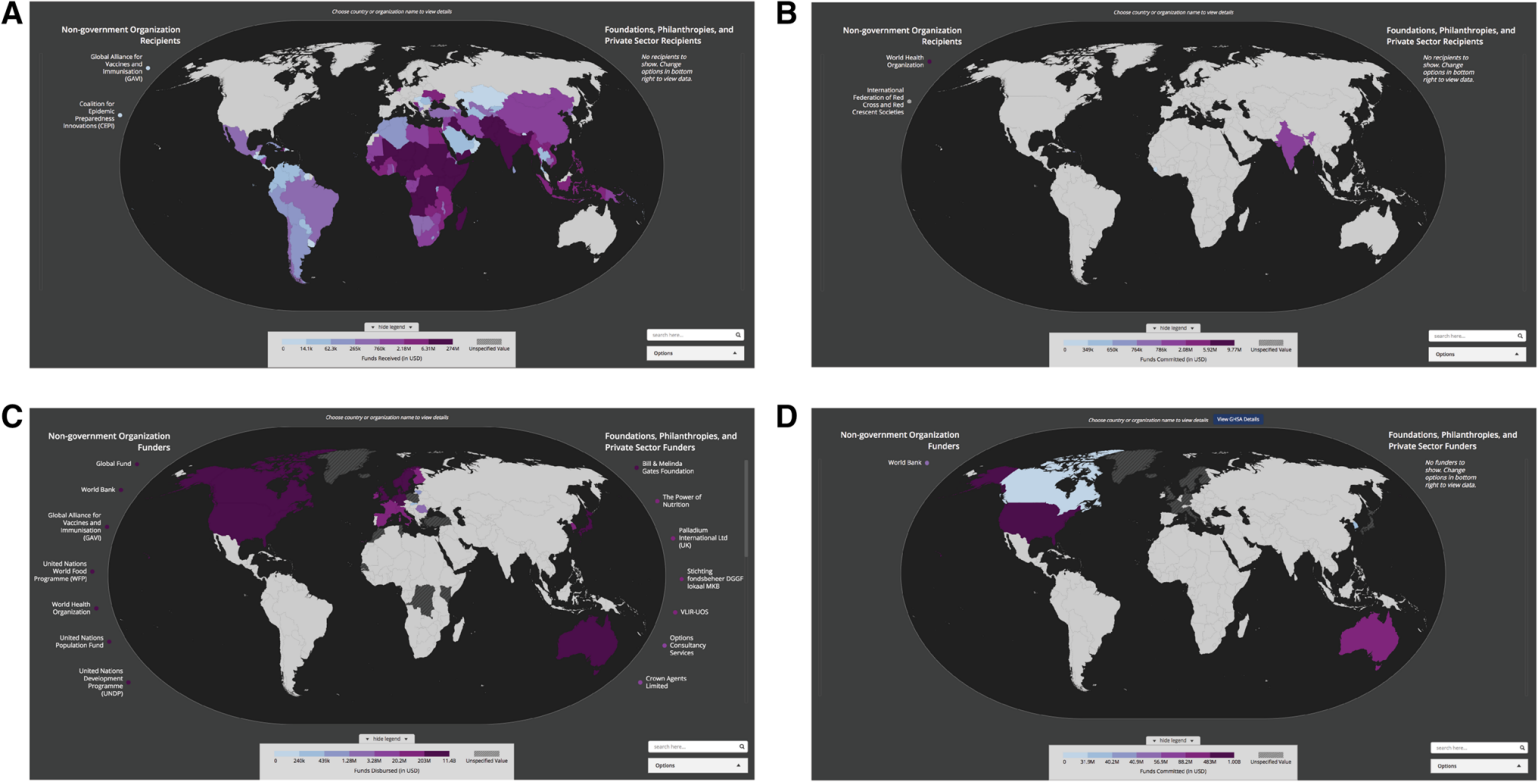
Global view of recipients of disbursed funds. **a** Global dashboard visualizing the amount of disbursed funds received per recipient country for immunization only. **b** Global dashboard visualizing the amount of disbursed funds received per recipient country for risk communication only. **c** Global dashboard visualizing the amount of disbursed funds funded per donor country for all core capacities. **d** Global dashboard visualizing the amount of committed funds per donor country under the Global Health Security Agenda.

The Global Health Security Tracking Dashboard is publicly available online at TRACKING.GHSCOSTING.ORG and provides a shared resource for both funders and recipients to understand and track who is funding what types of activities, where, in the context of global health security. While nearly all developing countries have received some level of funding for global health security, a global map of recipients highlights that funds are not equally distributed across geographic regions or across core capacities. For instance, while nearly global funding coverage exists for projects related to immunization (Fig. 1A), far fewer countries receive funds related to risk communication (Fig. 1B). The global view of funders (Fig. 1C) highlights that a relatively small number of funders support initiatives across many recipient nations and identifies the subset of those funders who disbursed funds specifically under the Global Health Security Agenda (Fig. 1D). These results identify areas of duplicative effort and gaps in funding to support GSA efforts.

**Figure 2:**
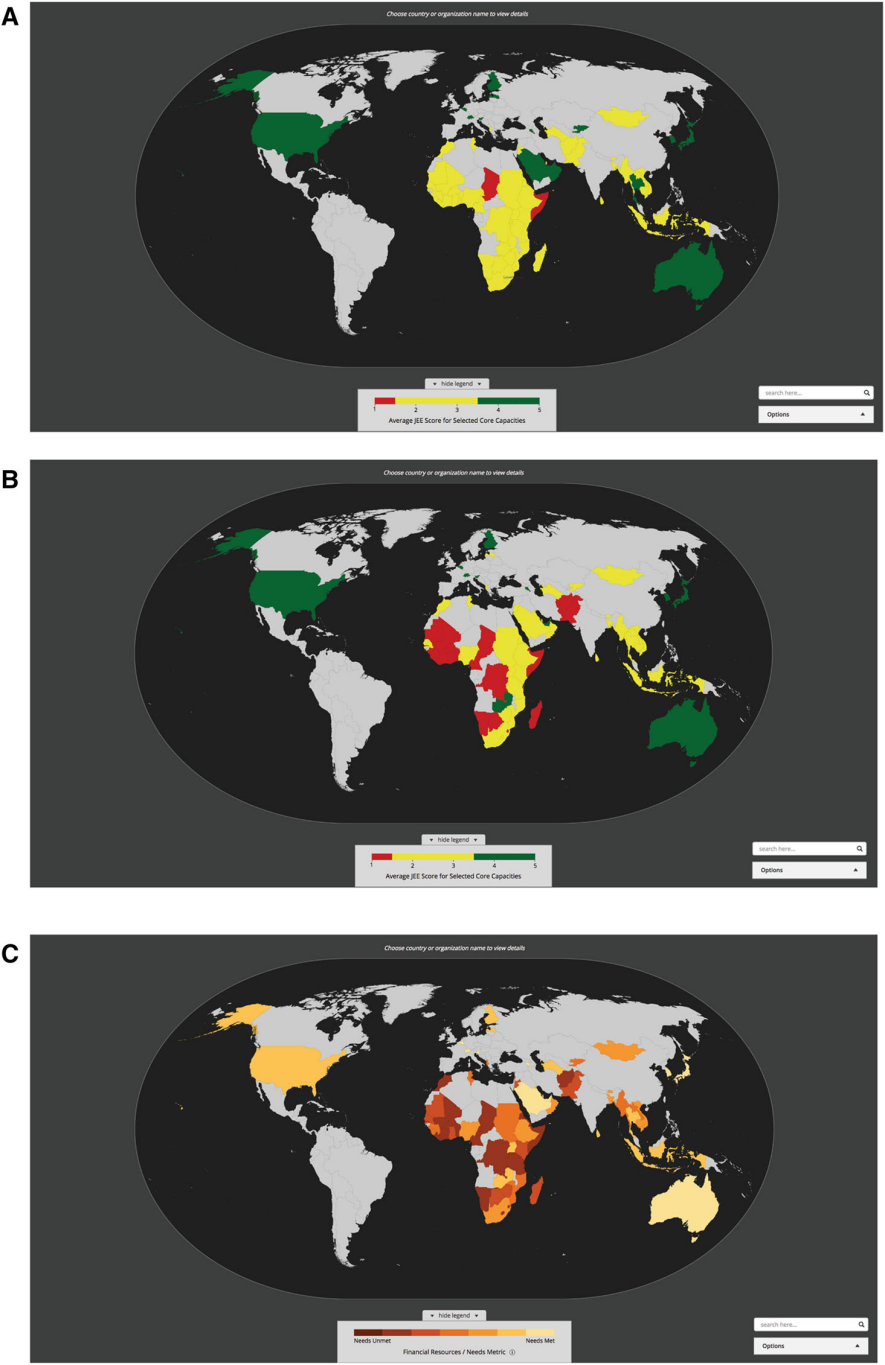
Global view of JEE scores and combined financial resources and needs metric. **a** Global view of overall JEE scores. **b** Global view of JEE scores for AMR. **c** Global view of combined financial resources and needs metric for AMR.

The 65 JEE evaluations completed and posted on the WHO Web site as of July 2018 demonstrate a broad range of capacities, across countries and technical areas, to prevent, detect, and respond to emerging infectious disease and other global health threats. The global distribution of JEE scores can be used to evaluate the need and prioritize the future investments (Fig. 2A). Visualizing need by JEE score across specific core capacities identifies those technical areas where capacity for each technical area is highest and lowest (Fig. 2B), and considering the ratio of financial resources to need highlights potential options for future funding targets based on funding history and existing capacity (Fig. 2C). For example, Mali received a score of *no capacity* for antimicrobial resistance (AMR), but received no reported disbursed funds for AMR from 2014 to 2018 despite the stated priority of AMR for many funders.

**Figure 3:**
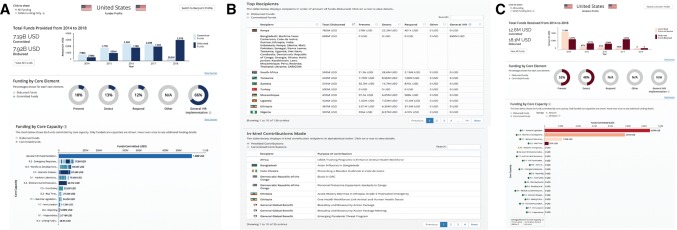
Funder and recipient profiles. **a** Funder profile characterizing the funds committed by the USA, over time, and by core capacity. **b** Funder profile identifying top recipients of funds disbursed from the USA, by core element, and identifying sortable list of in-kind contributions provided. **c** Recipient profile characterizing the funds received by the USA, over time and by core capacity.

Different funders provide financial resources and support to recipients across a broad range of geographic areas and core capacities. Visualizing a funder’s current commitments can help to highlight successes, as well as identify potential gaps in funding across regions or core capacities. For instance, the USA has committed significantly more funds for activities related to detection and response than for prevention, with particular support for medical countermeasures and personnel deployment (Fig. 3A), and has disbursed the most reported funds to Kenya, South Africa, and Tanzania (Fig. 3B). Recipient profiles identify the top funders and most funded core capacities relative to JEE scores, illustrating potential options for collaboration between funders and highlighting where needs align with funding efforts (Fig. 3C). Information pages for each funder and recipient provide project-level detail, highlight funding initiatives, track funding progress, and identify core capacities and regions that have received the most funds.

**Figure 4:**
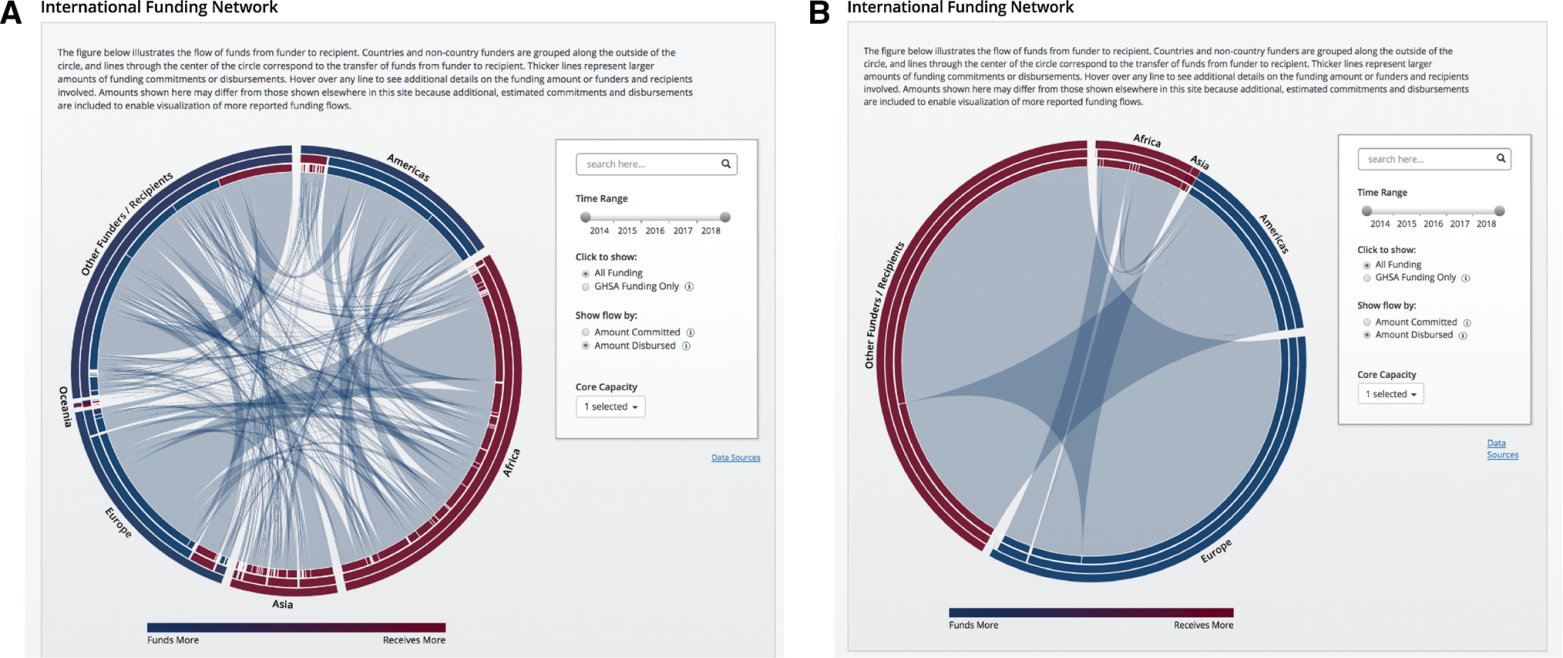
Global network of health security funding. The international funding network illustrates flow of funds between funders and recipients across time and core capacities. **a** International funding network for workforce development. **b** International funding network for biosafety and biosecurity.

Funding for global health security is part of a complex network of commitments and disbursements. This international funding network for global health security can be visualized based on the flow of funds from funders to recipients, characterizing the magnitude of financial resources transferred and the complexity of the global funding network. The number and size of transactions and the number of funders and recipients in this network vary across core capacities, highlighting those technical areas where the funding network is largest and more sustained as opposed to those that are small or reliant on a smaller number of funders. For instance, over 3000 reported funding commitments are identified to support workforce development, committed by over 45 funders to over 170 recipients (Fig. 4A), while fewer than 20 reported funding commitments, from significantly fewer funders and recipients, are identified to support biosafety and biosecurity (Fig. 4B). The international funding network graphic provides a visualization and communication platform to describe those areas where additional global coordination in health security funding could increase the strength of the funding environment by decreasing reliance on a small group of individual funders. The visualization provides a shared resource for communicating the value of such coordination by illustrating complementary funding activities occurring between countries, philanthropies, and private sector organizations.

## Discussion

The GHS tracking dashboard provides an interactive, publicly available, Web-based platform to support analysis by experts and non-experts in public policy, financing, global public health, and economic policy. This open source tool allows users to track and visualize information about international global health security funding through the use of a single data collection tool and interactive dashboard. Not only will the tool help define funding requirements based on existing gaps, but will also contribute to the development of compelling cases for investment, highlighting success stories and communicating need in regions.

We anticipate that the dashboard will promote a forum for mutual accountability, in which partners agree to be held responsible for the commitments they have voluntarily made to each other. The dashboard highlights opportunities for collaboration, with the goal of supporting a culture of mutual accountability and joint commitments to increasing GHS. The success of the dashboard, however, will require nations, philanthropies, and private sector donors to share their information, enabling funders to showcase successes, but also identify priorities for future investments.

## Electronic supplementary material

Below is the link to the electronic supplementary material.

Supplementary material 1 (DOCX 118 kb)

## Footnotes

1 Katz and Dowell (2015).

2 https://www.ghsagenda.org/docs/default-source/default-document-library/ghsa-legacy-report.pdf.

3 WHO (2016).

4 Gostin and Katz (2016).

5 http://www.un.org/News/dh/infocus/HLP/2016-02-05_Final_Report_Global_Response_to_Health_Crises.pdf.

6 https://foreignassistance.gov.

7 http://www.gavi.org/funding/donor-contributions-pledges/GAVI.

8 https://www.one.org/us/ebola-tracker/.

9 http://www.oecd.org/tax/automatic-exchange/common-reporting-standard/.

10 http://policycures.org/gfinder.html.

11 https://www.iatiregistry.org/.

12 https://extranet.who.int/spp/.

13 https://unstats.un.org/unsd/methodology/m49/.

## References

[CR1] Katz R, Dowell S (2015). Revising the international health regulations: call for a 2017 review conference. The Lancet Global Health.

[CR2] Advancing the Global Health Security Agenda: progress and early impact from U.S. investment. 2016 Available at: https://www.ghsagenda.org/docs/default-source/default-document-library/ghsa-legacy-report.pdf.

[CR3] WHO. *Sixty*-*ninth annual World Health Assembly: Implementation of the International Health Regulations (2005) Ebola virus disease*. (2016). Available at: http://apps.who.int/gb/ebwha/pdf_files/WHA69/A69_20-en.pdf

[CR4] Gostin L, Katz R (2016). The international health regulations: the governing framework for global health security. Milbank Quarterly.

[CR5] Protecting Humanity from Future Health Crises Report of the High-level Panel on the Global Response to Health Crises 25 January 2016. Available at: http://www.un.org/News/dh/infocus/HLP/2016-02-05_Final_Report_Global_Response_to_Health_Crises.pdf

[CR6] U.S. Government. Foreign Assistance Tracker. Available at: https://foreignassistance.gov

[CR7] GAVI. GAVI donor contributions. Available at: http://www.gavi.org/funding/donor-contributions-pledges/GAVI

[CR8] One Organization. Ebola Response Tracker. Available at: https://www.one.org/us/ebola-tracker/

[CR9] OECD. Common Reporting Standards, Available at: http://www.oecd.org/tax/automatic-exchange/common-reporting-standard/

[CR10] Policy Cures. G-Finder. Available at: http://policycures.org/gfinder.html

[CR11] International Aid Transparence Initiative. Available at: https://www.iatiregistry.org/

[CR12] WHO. Strategic Partnership Portal. Available at: https://extranet.who.int/spp/

[CR13] United Nations Statistics Division. Methodology: Standard country or area codes for statistical use (M49). Available at: https://unstats.un.org/unsd/methodology/m49/

