# Supplementary material for: Tracking the Flow of Funds in Global Health Security
Source: Ecohealth. 2019 Feb 28;16(2):298–305. doi: 10.1007/s10393-019-01402-w (PMC6682579; doi:10.1007/s10393-019-01402-w)
Supplement: Supplementary file 1 — Supplementary material 1 (DOCX 118 kb) [file 10393_2019_1402_MOESM1_ESM.docx]

# Technical Appendix

# Data sources

Supplemental table 1: Data sources and access

| **Data Source** | **Month accessed** | **Data from source incorporated into GHS tracking dashboard** |
| --- | --- | --- |
| International Aid Transparency Initiative (IATI) | June 2018 | Funding associated with 20 sectors (identified based on DAC-5 codes) relevant to IHR-related activities, or funding from the World Health Organization, for activities starting on or after January 1, 2014. Activities must have at least one non-zero transaction between 1 January, 2014 and 11 June, 2018. Data flagged by IATI as duplicates were excluded. |
| Article X Compendium | December 2017 | All funding and support identified in the Article X Compendium were incorporated into GHS tracking dashboard. |
| Nuclear Threat Initiative Commitment Tracker | May 2018 | All funding and support identified in the NTI Commitment Tracker were incorporated into GHS tracking dashboard, with the exception of commitments of countries to undergo the JEE evaluation process (within their own country). |
| WHO Contingency Fund for Emergencies | March 2018 | All funding and support announced in Geneva in March 2018 were incorporated into the GHS tracking dashboard. |
| Ebola Recovery Tracking Initiative | March 2018 | All funding and support identified in the Ebola Recovery Tracking Initiative were incorporated into the GHS tracking dashboard. |
| 2018 US White House GHSA Progress and Impact Report | March 2018 | All funding and support identified in the 2018 US White House GHSA Progress and Impact Report were incorporated into the GHS tracking tool, with the exception of funding or support that was duplicative of information already identified within the Article X Compendium. |

# Data structure

Supplemental table 2: Data elements and definitions

| **Data element** | **Description** | **Possible values** |
| --- | --- | --- |
| Data source | The source of the data reported in GHS tracking dashboard (e.g. IATI) | Any relevant data source |
| Project name | The name of the project, or a brief title describing the project’s purpose | Any project name |
| Brief project description | A brief description of the project and its purpose. Project descriptions are only included if they are directly provided by the data source being incorporated. | Any project description |
| Assistance type | Whether the assistance given was financial assistance or a form of in-kind support with no specific financial transaction. | *Financial Assistance:* transfer of funds directly from funder to recipient.  *In-kind support:* other forms of assistance that do not involve the direct transfer of funds from funder to recipient (e.g. deployment of personnel, transfer of goods and/or services). |
| GHSA funding or support | Whether or not the specified funds or support were provided under the Global Health Security Agenda (GHSA). Only funds specifically identified as GHSA funding or support by the entity reporting the funding are tagged as GHSA funding or support. | TRUE if assistance was committed or disbursed specifically under the GHSA; FALSE otherwise |
| Core capacities | The International Health Regulations (IHR) core capacity that the project directly supports, if any | Any set of core capacities corresponding to the first edition Joint External Evaluation (2005). |
| Funder name | The name of the funder | Any funder name |
| Funder country | The country of the funder (may be the same as “funder name” for government funders) | Any country |
| Recipient name | The name of the recipient of the funds or support | Any recipient name |
| Recipient country | The country of the recipient (may be the same as “recipient name” for government funders) | Any country |
| Transaction type | Whether support corresponds to a commitment or a disbursement. | *Commitment:* A commitment is an official obligation from a funder to provide a specified amount of funds or support for the benefit of the recipient.  *Disbursement:* Disbursed funds or support have been distributed to the recipient and/or placed at the disposal of the recipient. |
| Transaction years | The years during which the support was disbursed (or over which the commitment is intended to be disbursed) | Any year or range of years |
| Transaction amount | The amount of funds committed or disbursed | Any amount of funds |
| Transaction currency | The currency corresponding to the amount of funds committed or disbursed | Any currency specified based on an ISO 4217 Currency Code |

# Data processing and aggregation

The GHS tracking dashboard currently incorporates data from six different datasets, including the International Aid Transparency Initiative (IATI), the Article X Compendium, the Nuclear Threat Initiative Commitment Tracker, the Ebola Recovery Tracking Initiative, the WHO Contingency Fund for Emergencies, and data from the 2018 US White House GHSA Progress and Impact Report. Data from each of these sources were reviewed and processed in order to tag each project, where applicable, by core capacity, to ensure that data were in the necessary format, and to de-duplicate data between data sources. Note, as new data sources are identified, they are incorporated into the tool.

## Tagging GHSA funding and support

Selected data were identified as being funded under the Global Health Security Agenda (GHSA). All data from the 2018 US White House GHSA Progress and Impact Report and all data from the Nuclear Threat Initiative Commitment Tracker were identified as GHSA funding. In addition, selected efforts identified specifically as GHSA funding via the Article X Compendium were also identified as GHSA funding. No data captured via IATI, the WHO Contingency Fund for Emergencies, or the Ebola Recovery Tracking initiative were identified as GHSA funding.

## Tagging core capacities

When funding or support was provided to directly support one or more specific core capacities identified by the International Health Regulations, data were tagged with relevant core capacities. Due to their high volume, data from IATI were tagged with core capacities based on targeted string searches. For all other datasets, funding and support were tagged by core capacities based on review, by the research team, of the project name and description, and any additional information available in the report. Efforts that supported more than three specific core capacities, or that supported IHR implementation in general, were tagged as supporting “General IHR Implementation”.
